# Health Tract, No. 168

**Published:** 1863-09

**Authors:** 


### HEALTH TRACT, NO. 168.

### PHYSIOLOGY OF WORSHIP.

I HAVE come across men and women in my time, treading on the very verge of the grave in their old age, who were so eager after the making and saving of money; had become so close and stingy and mean-hearted in every thing pertaining to dollars and cents, that their whole character was overshadowed. Whatever of good there used to be in them had died out; they had but one god, and that was gold; and in thoughts of it they reveled; in talks of it they waked up into a newness of life, to a keenness of perception in every thing pertaining to number one, that at once astonished and surprised. All this was the result of the mind feeding itself, day by day and hour by hour, on thoughts of filthy lucre; and the propensity grew as any other would have done, had it been equally indulged in, until it became to be out of all proportion, gave a hue to the whole character, and the soul was lost in the love of gold,

"That vile idolatry."

In our physical nature, if any one set of muscles is exercised exclusively, they have an unnatural growth, approaching the monstrous, while others dwindle, and the whole physical nature is out of shape, uncomely, deformed. Hence those exercises most promote health of body which bring into play alternately every system of muscles. Thus it is also that if we exercise one set of muscles for a long time, we become weary, and yet may become rested without resting, and can return to the exercise with a feeling of freshness and new vigor, if for a while another set of muscles, or new combinations of them, are exercised. This principle pervades our moral nature as well; hence, for its proper nourishment, healthfulness, growth, and elevation, Divinity, for our best and highest good, has appointed one day in the week, and recommended in the Book of his revealed will a portion of the time of the other days in the week to be devoted to the contemplation of religious, of spiritual things; a proper attention to which breaks in upon the thoughts of worldliness, and effectually prevents that entire absorption of the mind as to money which makes old age so unlovely that we instinctively despise rather than revere. The whole subject merits the serious and solemn consideration of every reader, as there is not one who is not in danger of the great calamity of wrecking the very soul in its greed of gold, of becoming a monomaniac, and an object of pity and contempt to all. The value of this habit of daily contemplation and of stated weekly meditation on things which pertain to our spiritual nature and its relations to God and eternity, is seen in the old age of individuals who are evidently ripe for heaven; and in whole communities, as the Society of Friends, one of the cardinal points in whose religious faith is, the duty of self-communion, of inward spiritual contemplation; and that this is profitable to soul and body; for "the life that now is, and that which is to come," witness their placid nature, their thriving condition, and the statistical fact that their lives average ten or fifteen years longer than any other class of persons.

